# Depletion of CTCF disrupts PSG gene expression in the human trophoblast cell line Swan 71

**DOI:** 10.1002/2211-5463.13087

**Published:** 2021-03-02

**Authors:** Da Som Jeong, Myoung Hee Kim, Ji‐Yeon Lee

**Affiliations:** ^1^ Department of Anatomy Embryology Laboratory Yonsei University College of Medicine Seoul Korea; ^2^ Brain Korea 21 PLUS project for Medical Science Yonsei University College of Medicine Seoul Korea

**Keywords:** CTCF, epigenetic modification, pregnancy‐specific glycoproteins, Swan 71, trophoblast

## Abstract

Pregnancy‐specific glycoproteins (PSGs) are fetal proteins secreted by the placenta during pregnancy. The PSG level in maternal serum is an indicator of risk for pregnancy complications. However, little is known about the molecular mechanisms underlying PSG gene expression. Recently, the importance of epigenetic regulation of placental genes has been emphasized in the study of developmental defects and placental disease. In this study, the role of the CCCTC‐binding factor (CTCF) in regulation of PSG expression was investigated to better understand the epigenetic regulatory mechanisms of the PSG genes. Inhibition of CTCF expression disturbed transcription of several PSG genes: *PSG1*, *PSG2*, *PSG4*, *PSG5*, *PSG8*, and *PSG9* were upregulated and *PSG6* and *PSG11* were downregulated. These transcriptional changes were correlated with decreased CTCF binding and changes in histone modification at the PSG promoters. Our data demonstrate that CTCF is a potential mediator in the regulation of PSG gene expression.

## Graphical abstract

The transcriptional factor CCCTC‐binding factor (CTCF) is involved in the regulation of clustered genes. We investigated whether CTCF could regulate the pregnancy‐specific glycoprotein (PSG) family genes associated with successful pregnancy. Interestingly, the effects of CTCF silencing on the expression of PSG genes are not identical and appear to act individually for each gene. These transcriptional changes are correlated with histone modifications.

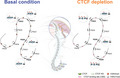

**Abbreviations**
CEA: carcinoembryonic antigen
ChIP: chromatin immunoprecipitation
CTCF: CCCTC‐binding factor
H1‐hESC: H1 human embryonic stem cell
HUVEC: human umbilical vein endothelial cell
HVMF: human villous mesenchymal fibroblast
IUGR: intrauterine growth retardation
PE: pre‐eclampsia
PSGs: pregnancy‐specific glycoproteins
UCSC: University of California Santa Cruz

Pregnancy‐specific glycoproteins (PSGs) are the members of the carcinoembryonic antigen family, one of immunoglobulin super families. PSG genes are clustered on chromosome 19q13.2 encoding 10 genes in humans and chromosome 7 comprising 17 genes in mice [1]. Human PSGs are expressed in the syncytiotrophoblast of the placenta and secreted into the maternal bloodstream. PSGs are among the most abundant fetal proteins detected in the maternal circulation produced by the placenta during pregnancy [2]. The known functions of PSG during pregnancy are implicated in immune regulation, angiogenesis, and platelet regulation, which are important for normal placental development [3, 4, 5]. Defects in placental development are directly associated with the disruption of the maintenance of pregnancy [6]. In particular, several case–control studies have shown that low levels of PSG are associated with complicated pregnancy outcomes such as intrauterine growth retardation (IUGR), preterm labor, and pre‐eclampsia (PE) [7, 8, 9]. These reports imply the pivotal roles of PSG in successful pregnancy outcomes; however, the molecular mechanism underlying the regulation of PSG gene expression is not well understood.

During development, epigenetic changes modulate the accessibility of transcription factors to the DNA and consequently affect gene expression. Epigenetic mechanisms during development include DNA methylation, histone modifications, the action of non‐coding RNAs, and 3D structural changes in the chromatin, all of which affect cell phenotype and function and contribute to the associated pathological conditions [10]. Many recent studies have shown that epigenetic modifications are related to placental development and disease during pregnancy [11, 12, 13, 14]. Appropriate placentation and fetal environment are not only important for the intrauterine growth and survival of the fetus but also affect neurodevelopmental disease and metabolism in the postnatal phase through a process called fetal reprogramming [15]. Under pathologic conditions such as PE, IUGR, and small for gestational age, epigenetic dysregulation correlates with abnormal placental gene expression [16]. It is, therefore, imperative to understand the expression pattern of placental genes and their epigenetic regulatory mechanisms to elucidate the causal relationship between placental development and disease during pregnancy and for the development of preventive measures or treatment regimens.

Among the epigenetic modifiers, the CCCTC‐binding factor (CTCF) is a highly conserved, and ubiquitously expressed DNA‐binding protein. CTCF regulates gene transcription as an activator, a repressor, or an insulator and serves as a core chromatin organizer by contributing to establishing the three‐dimensional structure of the genome [17]. Abnormal CTCF binding to the DNA‐binding sites may perturb appropriate long‐range chromatin interactions, thereby regulating genomic instability [18]. Many studies have shown that abnormal CTCF binding may cause disturbances in the transcriptional regulation of tumor suppressors or oncogenic genes in many human cancers. CTCF also regulates embryonic development by affecting the expression of several genes that influence cell survival and proliferation (reviewed in [19]). Specifically, CTCF has been demonstrated to regulate the clustered gene families such as HOX, protocadherin, and β‐globin through the organization of chromatin structures [20, 21, 22]. DNA methylation, which is involved in a wide range of gene expression during development and disease, is known to affect the CTCF‐DNA binding occupancy [23]. A study showing the disruption of endothelial vascular development in the placenta of CTCF‐deficient mice demonstrates the importance of CTCF for adequate placentation to facilitate normal fetal development [24].

To date, no study has reported the association between CTCF and regulation of PSG gene expression. In the present study, we investigated whether CTCF binding, which is highly conserved across the PSG loci, contributes to the regulation of PSG gene expression in trophoblast cells. Further understanding of the mechanisms underlying PSG gene regulation will lay the scientific foundation for the development of diagnostics and therapeutics for placental disease.

## Materials and methods

### Cell culture and transfection

The human extravillous trophoblast cell line Swan 71 was kindly provided by J.‐Y. Kwon (Institute of Women's Life Medical Science, Yonsei University Health System, Seoul, Korea). These cells were cultured in Dulbecco's modified Eagle's medium (WELGENE Inc., Daegu, Korea) supplemented with 10% FBS (WELGENE Inc.), 1× antibiotic‐antimycotic solution (WELGENE Inc.), 1× HEPES buffer solution (WELGENE Inc.), and 1× non‐essential amino acid solution (WELGENE Inc.) in humidified air at 37 °C and 5% CO_2_. For gene knockdown (KD) study, Swan 71 cells were seeded at 1 × 10^6^ cells in 100 mm culture plate and were transfected with 10 nm of siRNA using G‐fectin (Genolution, Seoul, Korea) for 48 h. ON‐TARGET plus CTCF siRNA (L‐020165‐00‐0005) were purchased from Dharmacon (Cambridge, UK), which utilizes a patented dual‐strand modification to reduce off‐target effects. Negative control siRNA (sense sequence, 5′‐CCUCGUGCCGUUCCAUCAGGUAGUU‐3′; antisense sequence, 5′‐CUACCUGAUGGAACGGCACGAGGUU‐3′) was provided by Genolution Inc.

### Total RNA isolation and reverse‐transcription PCR

Total RNA from cultured cells was extracted using Trizol reagent (Invitrogen, CA, USA) according to the manufacturer's instructions. cDNA synthesis was performed with 1 μg of total RNA using ImProm‐II Reverse Transcriptase (Promega, Madison, WI, USA) and RNase inhibitor (Promega). Since the nucleotide sequence of all PSG genes is very similar, the primer design process and PCR conditioning were carefully adjusted to guard against off‐target priming. PCR conditions for amplification of PSG cDNAs were as follows. An initial denaturation step was done at 95 °C for 10 min, followed by 30–42 cycles of denaturation at 95 °C for 40 s, annealing at 58 °C for 20 s, and extension at 72 °C for 30 s. The final extension step was done at 95 °C for 5 min. After confirming that the PCR products of all PSG genes appeared as a single band with the correct size, RT‐qPCR was performed. For RT‐qPCR, cDNA was amplified using SYBR Green PCR Master Mix (Applied Biosystems, Carlsbad, CA, USA) on an ABI7300 real‐time PCR system (Applied Biosystems). PCR primers are listed in Table 1.

**Table 1:** Primer sequences for RT‐PCR and ChIP‐PCR.

Primer sequences for qRT‐PCR		
	Forward primer (5′–3′)	Reverse primer (5′–3′)
PSG1	GAG CTT GAG AAT TGC TCC TGC	GAG GGG CTG AGA GGG TTC
PSG2	CCA CCC ATG AGC CTG GGA AT	CCA GGA GCC CCT TCC ATT TGA TGT
PSG3	GGC AGA CAG TTG CTT TCA TTC TT	TTC TGG GGC ACT TAG GGA GC
PSG4	CAT GTG AGC CAC TCA GAA CTC A	TAA GAG GGG TGG GAG CCT TA
PSG5	CCT GGA GAG AGG CTC AGC A	CAG GGC TTC AAT CGT GAC TTG
PSG6	ACT TAA ACC CCA GGG AGA AG	GTT TCC ATG GCA GGG ACC A
PSG7	TCC GTG ACA GTC AGA GTC TC	CTG ACT TAT AGG GCT TCT GG
PSG8	CAA GCC CTA CAT CAC CAT CAA C	GAT GCC ACC ATA TTG GTC CCT T
PSG9	TCC TGC ACA CAG CGC ATC	GGT GTA GGT TCC TGC ATC CTT
PSG11	GTC TCA GCG CAG AAG GAG G	CCA TTT GAT GTG CTC TGT GC
β‐actin	CAT GTT TGA GAC CTT CAA CAC CCC	GCC ATC TCC TGC TCG AAG TCT AG

Primer sequences for ChIP‐PCR			
	Forward primer (5′–3′)	Reverse primer (5′–3′)	Position from TSS
PSG1‐up	TGG AAT TGC TGC CGT TCA CAC	TCA CGA GGG GAA AGC ACC CG	−1427 to − 1751
PSG2‐up	GAG GTC AGG AGG GGA AAG CT	GTG TGA AGT GTG CAG ATC CAC A	−1361 to − 1755
PSG3‐up	GCT GGA AGG TGA AGG AGC TA	TTA GGG AGC AGT GAC AGA AC	−1354 to − 1827
PSG4‐up	AGG TCA CGA GGG GAA AGC GT	GTC ACA CAG ACT GGG ACT CAA	−1279 to − 1737
PSG5‐up	AGG AGC AGG TGT GTG GGG CT	CGT GTG AAG TGT GCA GAT CCA T	−1464 to − 1777
PSG6‐up	GAG CTA CCT GCA GAG GGC	CTG CCT TTG GGA CAT CTG AC	−1493 to − 1900
PSG7‐up	CAG CTC TAG GCT TCA CCT GC	ACA CTT CCC TCC CTG TCC AA	−1344 to − 1776
PSG8‐up	CAC GAG GGG AAA GCG CCT	CGT GTG AAG TGT GCA GAT CCA T	−1461 to − 1850
PSG9‐up	GAC CTC GTG TGA AGT GGA ATA A	GGA AGG AGC AGG TGT GTG G	−1486 to − 1793
PSG11‐up	AGG AAC TAC CTG TAG GGG GA	GTG AAG TGT GCA GAT CCA CGT A	−1520 to − 1811
Gene desert	TGT TTT TGC AGT TAC TAG ACT GGG	TT CCC CTG CTT TTA TGT GG	

### ChIP assay

Swan 71 cells were harvested at 80–90% confluency with 0.7 × 10^6^ cells per antibody. The cells were cross‐linked with 1% formaldehyde using 37% formaldehyde for 15 min at room temperature. To quenching the reaction, 2.5 m glycine was added to make 125 mm for 10 min at room temperature. Then, cells were washed twice with 1× cold PBS and lysed in lysis buffer for 10 min on ice. Lysis buffer consisting of 1% SDS, 1% Triton X‐100, 0.1% sodium deoxycholate, 10 mm EDTA, 50 mm Tris–HCl (pH 8.0), and a protease inhibitor (Merck, Darmstadt, Germany) was added. Sonication sheared 500–1000 bp DNA fragments, which is confirmed by loading with 1% agarose gel. Then, the lysates were incubated with Protein A/G PLUS‐Agarose (Santa Cruz, Dallas, TX, USA) in a rotator for 1 h at 4 °C. For DNA‐protein complexes, precleared DNA is incubated with antibody overnight at 4 °C. ChIP‐grade anti‐CTCF antibody (Cell Signaling Technology, Beverly, CA, USA), normal rabbit IgG (Santa Cruz), anti‐PolⅡ (Santa Cruz), anti‐histone H3 trimethyl K4 (Abcam, Cambridge, UK), and anti‐histone H3 trimethyl K27 (Abcam) were used.

### ChIP‐seq database

**Fig. 1:**
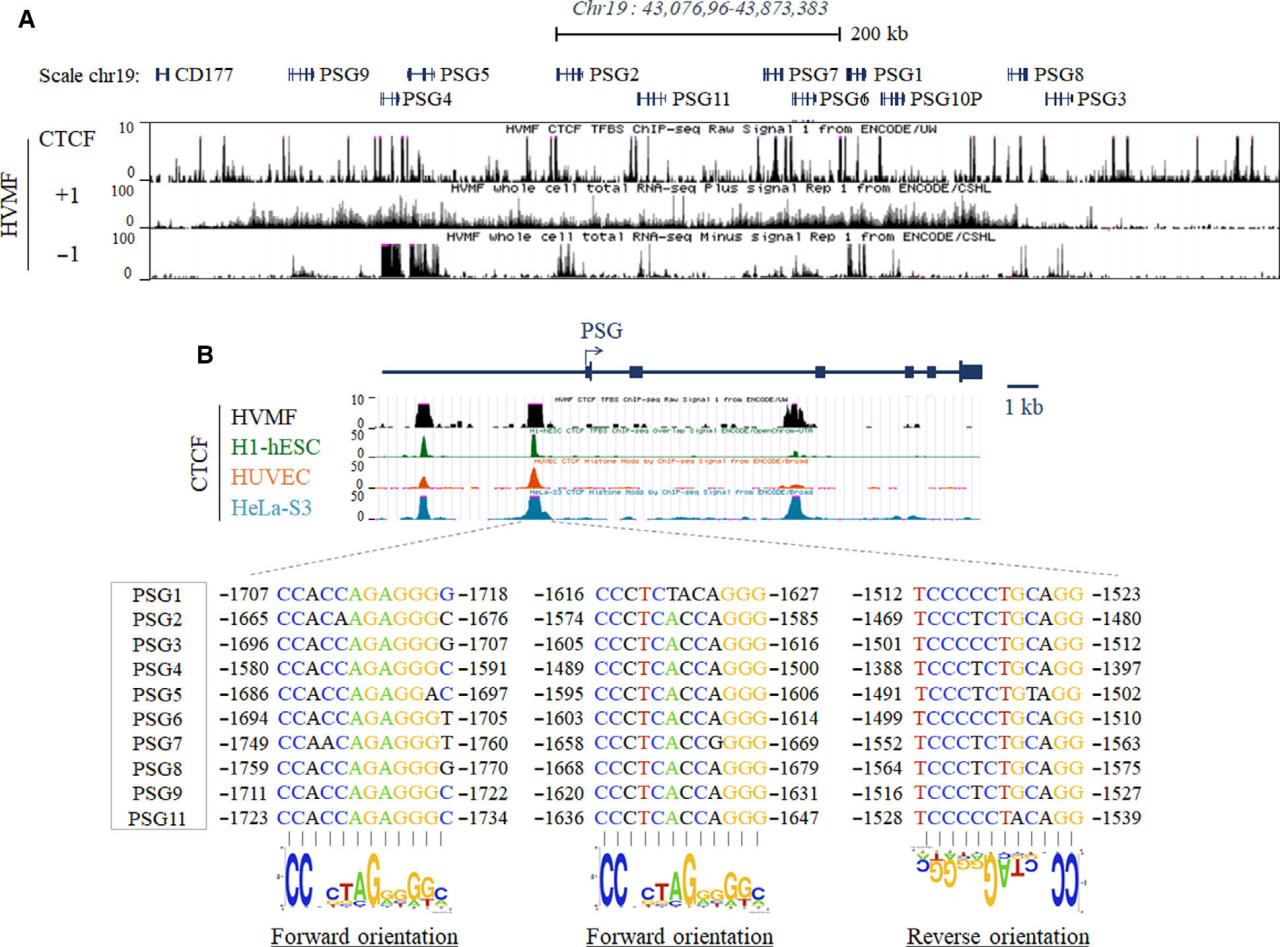
Conserved CTCF occupancy in several different cell lines across the PSG gene loci. (A) Schematic representation of the PSG locus containing the CBSs and amplicon sites in the cluster. A screen capture of the UCSC genome browser that shows the CTCF binding peaks and RNA‐seq signals of HVMF cell line in the genomic region of chr19: 43 076 96–43 873 383 that contained the PSG genes. (B) CBSs with consensus sequences in the region 1.6–1.8 kb upstream of each PSG gene in various cell types (HVMF, H1‐hESC, HUVEC, and Hela‐S3). Of the three consensus CTCF binding motifs, two showed forward orientation and the one closest to the TSS had reverse orientation. Three consensus binding motifs showed a similar percentage of conservation.

Bioinformatic analyses for CTCF binding sites (CBSs) were performed using the University of California Santa Cruz (UCSC) genome browser (https://genome.ucsc.edu/). The ChIP‐seq data for four cell lines represented in Fig. 1A can be accessed using the following codes: HeLa‐S3, GSM749729; h1 human embryonic stem cell (H1‐hESC), GSM733672; human umbilical vein endothelial cell (HUVEC), GSM749749, and human villous mesenchymal fibroblast (HVMF), GSM1022630.

### Statistical analysis

Data were obtained from three separate experiments and represented as mean ± standard error of the mean (SEM). The Student's *t*‐test was used for comparison between siCTCF‐treated cells and control. Differences were statistically significant at *P* < 0.05.

## Results and Discussion

The binding of CTCF to the DNA is determined by the recognition of the consensus sequence; some binding sites exhibit differential CTCF binding patterns depending on the tissues, while others consistently show strong binding to CTCF in most tissues. CBSs distribute all throughout the genome, that is, intergenic region, gene bodies, and promoters, and its role is determined by where the CTCF binds [25]. Before exploring the role of CTCF as a PSG gene regulator in trophoblast cells, we first examined the CTCF binding patterns across the PSG gene loci using publicly available data. Among the experimental models from which CTCF ChIP‐seq data deposited in ENCODE were derived, HVMF is the only placenta‐related cell line. A view of chromosome 19 containing PSG genes (chr19: 43 076 96–43 873 383) shows numerous CTCF binding signals in many regions of the PSG gene body, as well as in the upstream and downstream regions (Fig. 1A). RNA‐seq data show that several PSG genes are expressed in the placental HVMF cell line. In the enlarged genomic map, three strong CTCF binding peaks are common to all PSG genes, except *PSG3* and *PSG8*, two of which are in the upstream region of each gene (Fig. 1B). In addition to HVMF, these peaks are highly conserved in various cell types, including HUVEC, embryonic stem cells, and Hela cells. One is located 1.6–1.8 kb upstream, and the other is located 5.5–5.7 kb upstream from the transcription start site (TSS) of each PSG gene. In the case of *PSG3* and *PSG8*, only one peak close to the TSS is observed. Therefore, we focused on the proximal peak close to the TSS observed in common in all PSG genes and studied the location, orientation, and conservation status of CBSs. As a result, we found three consensus binding sequences for all 10 PSG genes, two being forward orientation motifs and one being reverse orientation motif.

**Fig. 2:**
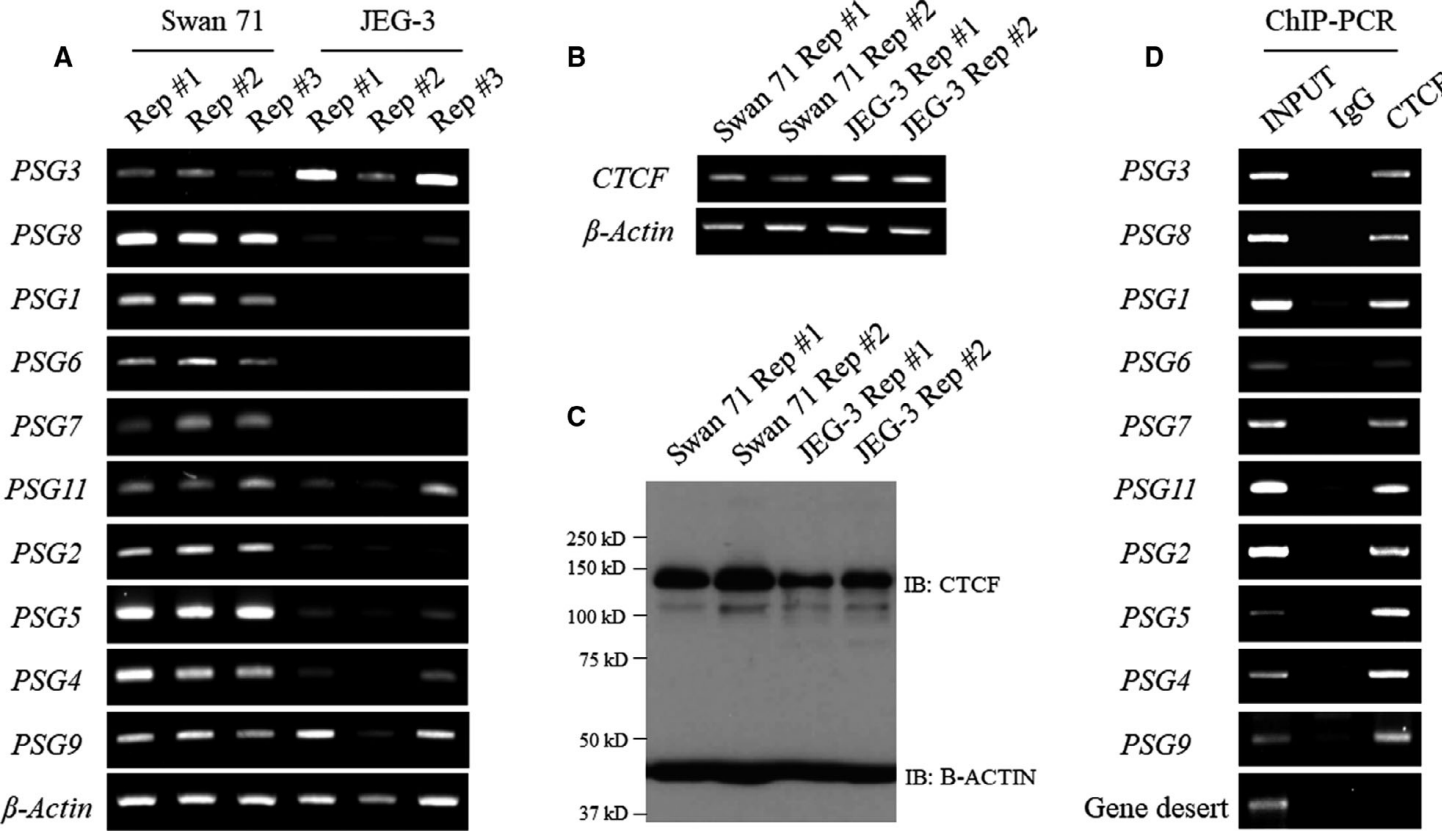
The expression of PSG and CTCF in placental cells. (A) Comparison of PSG gene expression in Swan 71 and JEG‐3 cells by RT‐PCR. β‐Actin was used as an internal control for RT‐PCR. (B) mRNA levels of CTCF in human placental cell lines. The number of each sample indicates two biological replicates. (C) Protein levels of CTCF in human placental cell lines. (D) ChIP‐PCR results for CTCF binding at the designed amplicon sites in Swan 71 cells, which express the entire set of PSG genes. Primers for gene desert regions (chr20:57 828 313–57 828 465) were used as negative control for ChIP‐PCR.

To investigate the role of CTCF in the regulation of PSG gene expression, we needed an appropriate cell line model in which all PSG genes were expressed, so first analyzed the mRNA expression of 10 PSGs (*PSG1‐PSG9, PSG11*) from two different human trophoblast cell lines, Swan 71 and JEG‐3, by RT‐PCR. Unlike JEG3, the Swan71 cells were found to express the entire set of PSG genes under normal conditions (Fig. 2A), indicative of their applicability as an excellent model system to study the role of specific factors affecting gene expression at the site of PSG gene. Although the expression of several PSG genes has been previously identified in other trophoblast cell line and primary placental cells [26, 27], no studies have demonstrated the expression of all human PSG genes in a particular cell line or tissue as shown in this study. On the other hand, CTCF, known as a ubiquitously expressed architectural protein, is well expressed in both trophoblast cell lines, JEG‐3 and Swan 71 (Fig. 2B,C). Therefore, the ChIP experiment to examine CTCF occupancy in the PSG gene locus was performed using Swan 71 cells in consideration of the expression pattern of the PSG genes. Based on the consensus sequence motif shown in Fig. 1B, we designed amplicons for ChIP‐PCR to cover three binding sites in the upstream region of each PSG gene. PCR primer sequences used to amplify each amplicon site are listed in Table 1. ChIP‐PCR results showed that CTCF binds to the upstream region of all PSG genes from *PSG1* to *PSG11* throughout the locus (Fig. 2D).

**Fig. 3:**
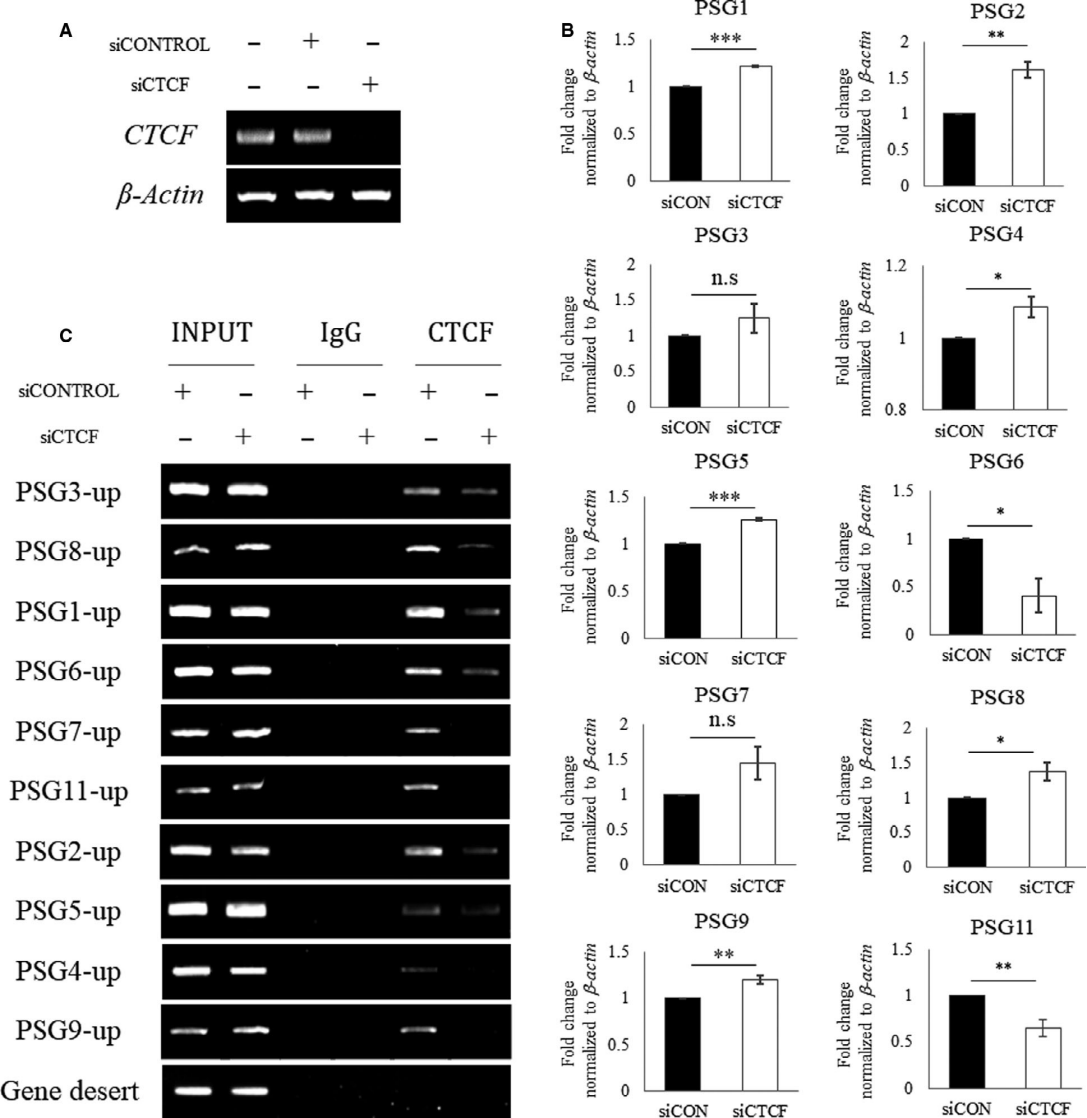
Effect of CTCF depletion on PSG gene expression in Swan 71 cells. (A) CTCF expression levels in siRNA‐treated cells. (B) Fold change in the expression level of PSG genes upon CTCF KD. The expression of each PSG indicates the relative level of the *C*_t_ value of the siCTCF to the *C*_t_ of the siCON. All data were obtained from three separate experiments, and differences were represented as mean ± SEM. The Student's *t*‐test was used for comparison between siCTCF and siCON. Significant differences were considered as *P < 0.05, **P < 0.01, ***P < 0.001. (C) ChIP‐PCR results showing the decrease in CTCF binding at the proximal region of PSG genes following CTCF expression KD.

To gain further evidence on whether CTCF contributes to the transcription of the PSG gene cluster, the mRNA level of each PSG gene was measured following the silencing of CTCF expression. The siRNA‐mediated KD of CTCF significantly downregulated CTCF expression (Fig. 3A). In this condition, the mRNA levels of PSG genes were affected; some PSG genes, such as *PSG1*, *PSG2*, *PSG4*, *PSG5*, *PSG8*, and *PSG9,* were upregulated, while *PSG6* and *PSG11* genes were downregulated (Fig. 3B). Under the CTCF KD condition, CTCF binding was significantly reduced at all PSG gene loci tested (Fig. 3C). These results suggest that CTCF binding to the upstream region of PSG genes is involved in the transcriptional regulation of PSG genes. Our data also show that CTCF binding exerts different effects on individual PSG genes. In other words, the loss of CTCF binding upregulates the expression of some PSG genes, while downregulating the expression of others.

**Fig. 4:**
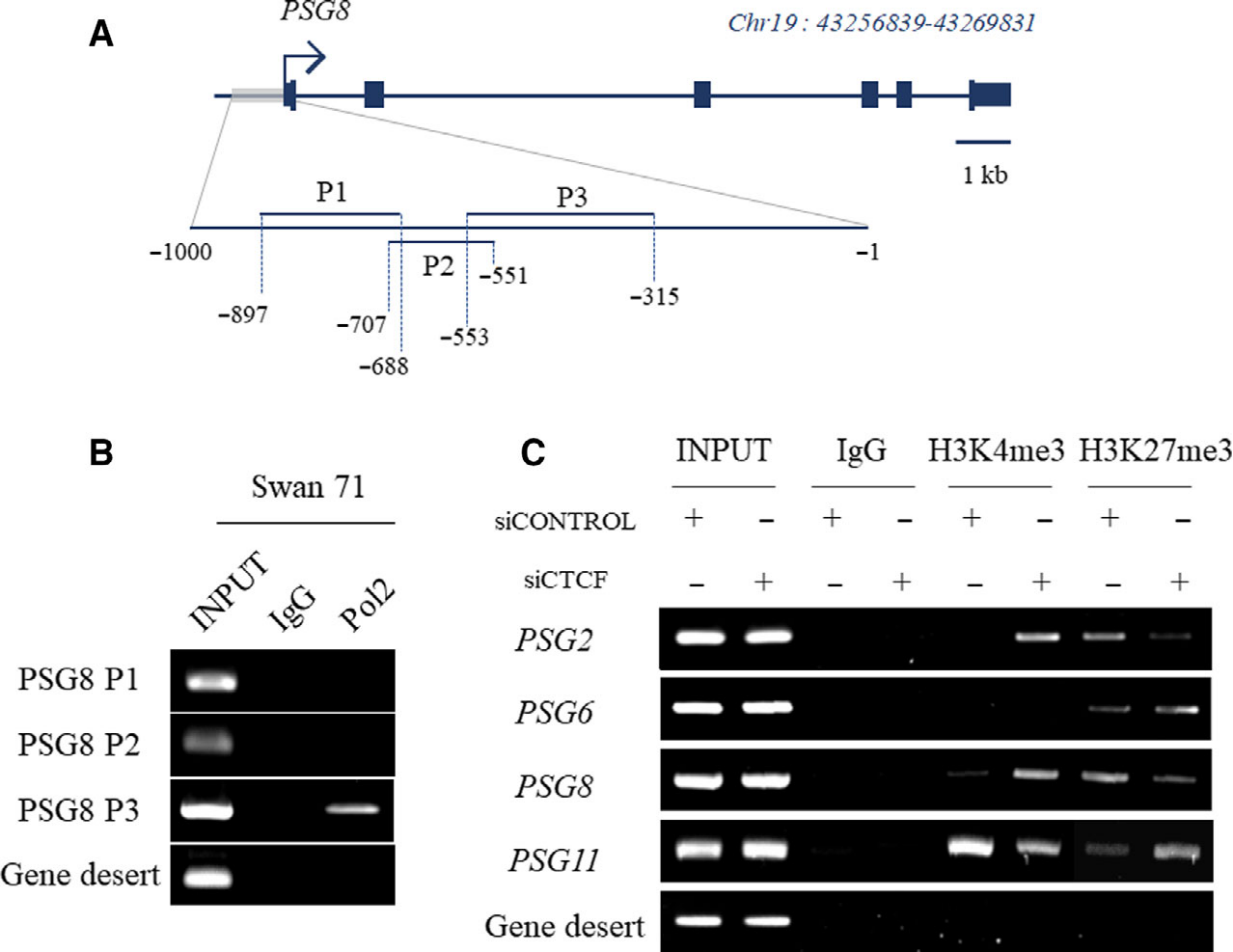
Histone modification changes in the promoter of PSG genes in response to CTCF expression KD. (A) Genomic map of *PSG8* and amplicons for the detection of Pol2 binding site. The 1 kb long‐range putative promoter region was divided into three sites as follows: P1 (−688 to −897), P2 (−551 to −707), and P3 (−315 to −553). (B) ChIP‐PCR result showing the strongest Pol2 binding at P3 site. Amplicon for gene desert region was identical to that used in Fig. 2D. (C) Changes in the enrichment of H3K4me3 active histone mark and H3K27me3 repressive mark at the PSG gene promoter following CTCF expression KD. To determine the binding pattern of each histone mark, three biological replicates were generated and analyzed.

Next, to examine whether the CTCF KD‐mediated variation in PSG expression accompanies epigenetic changes, we analyzed the modification patterns of histone proteins enriched in the upstream regions of PSG genes. As the promoter region of PSG genes is not clearly defined from the current literature, we tried to narrow down the scope of the interesting region by evaluating the polymerase II (Pol2) binding position. Three amplicon sites were generated by dividing the 1 kb upstream region from the TSS site of each PSG gene. *PSG8* is presented as a representative gene (Fig. 4A). PCR was performed using DNA obtained by performing ChIP experiment with Pol2 antibody. Of these sites, the P3 site showed the strongest binding to Pol2 (Fig. 4B). Therefore, we analyzed for H3K4me3 and H3K27me3 enrichment, targeting approximately the same region in several other PSG genes, including *PSG8*. *PSG2* and *PSG8* were selected as the genes upregulated after CTCF KD, while *PSG6* and *PSG11* were chosen as the downregulated genes. The increase in the level of *PSG2* and *PSG8* transcripts after CTCF silencing was accompanied by an increase in the recruitment of active mark H3K4me3 and a decrease in the recruitment of repressive mark H3K27me3. The downregulation of *PSG6* and *PSG11* expression mediated by CTCF KD was consistent with the enrichment of H3K27me3 rather than reduction of H3K4me3 (Fig. 4C).

Together, our results provide evidence that CTCF may regulate PSG gene expression via epigenetic mechanisms. Keeping in mind that CTCF will define the topological boundary of chromatin, CTCF deficiency is expected to cause expression changes in the same direction for all genes in the cluster, but our data show that there are individual genes that are more particularly affected by CTCF deficiency. This suggests that CTCF is likely to cooperate or counteract with other transcription factors or cofactors bound to specific gene sites rather than acting alone when involved in gene transcription. Considering that functional changes are induced upon the manipulation of PSG expression, as shown in previous studies [28], PSG deregulation by CTCF shown in the present study may lead to dysfunction of PSG. On the other hand, apart from the effect of CTCF expression level, a change in DNA methylation pattern can affect the ability of CTCF to bind to the target gene. During development, the DNA methylation status of the placenta is the least methylated unlike other tissues [29, 30, 31]. Abnormal regulation of placental DNA methylation is associated with pregnancy disorders [32, 33, 34, 35].

Thus, changes in DNA methylation patterns and dysregulated CTCF expression can both affect the expression of the PSG genes at developmental stages or especially in complicated pregnancy. As mentioned earlier, PSG is considered an important factor in maintaining pregnancy based on several previous studies showing its role in placental development, vascular morphogenesis, and immunomodulation. While the specific function of each PSG gene or its regulatory mechanism is not yet known, our results demonstrating the role of CTCF as a regulatory factor of PSG genes may take one step closer to revealing the unknown field.

The next step in understanding how CTCF contributes to structural changes in chromatin at the PSG locus will be to explore the promoter and enhancer regions involved in PSG gene transcription and further investigate the changes in looping structure by CTCF. Accumulation of relevant studies may answer whether the CTCF‐mediated spatial chromatin organization contributes to the normal expression of the placental PSG clustered gene family during pregnancy.

## Conclusion

Our results showed that CTCF, which is known to regulate the transcription of several clustered genes, also may regulate the transcription of human PSG genes (*PSG1*‐*PSG9*, *PSG11*) in trophoblast cells. Silencing of CTCF expression upregulated or downregulated the expression of several PSG genes, and this effect was accompanied by epigenetic changes. H3K4me3 was enriched in the promoter region of the upregulated PSG genes, whereas H3K27me3 was recruited to the promoter region of the genes with reduced transcripts, such as *PSG6* and *PSG11*. Further studies are warranted to determine whether CTCF mediates the communication between PSG genes and regulatory elements and induces any alterations in the chromatin structure. In conclusion, our findings suggest that CTCF may be involved in the transcriptional regulation of PSG genes once its expression is induced and maintained in the placenta during pregnancy.

## Author contributions

DSJ and J‐YL designed the experiments, analyzed the data, and wrote the manuscript. MHK and J‐YL supervised the study and finalized the manuscript.

## Conflict of interest

The authors declare no conflict of interest.

## Data Availability

Data will be available from the corresponding author upon reasonable request.
